# SP1 transcriptionally activates HTR2B to aggravate traumatic spinal cord injury by shifting microglial M1/M2 polarization

**DOI:** 10.1186/s13018-024-04678-z

**Published:** 2024-04-08

**Authors:** Qifei Xu, Fanguo Kong, Guanghui Zhao, Junwei Jin, Shengkai Feng, Ming Li

**Affiliations:** 1 Department of Orthopedics, The First People’s Hospital of Pingdingshan, Pingdingshan, 467000 China; 2 Department of Orthopedics, Henan Provincial Orthopedic Hospital, No. 100, Yongping Road, Zhengdong New District, Zhengzhou, 450045 China

**Keywords:** Spinal cord injury, SP1, HTR2B, Microglia, Macrophage polarization

## Abstract

**Background:**

Spinal cord injury (SCI) can result in structural and functional damage to the spinal cord, which may lead to loss of limb movement and sensation, loss of bowel and bladder control, and other complications. Previous studies have revealed the critical influence of trans-acting transcription factor 1 (SP1) in neurological pathologies, however, its role and mechanism in SCI have not been fully studied.

**Methods:**

The study was performed using mouse microglia BV2 stimulated using lipopolysaccharide (LPS) and male adult mice subjected to spinal hitting. Western blotting was performed to detect protein expression of SP1, 5-hydroxytryptamine (serotonin) receptor 2B (HTR2B), BCL2-associated x protein (Bax), B-cell lymphoma-2 (Bcl-2), inducible nitric oxide synthase (iNOS), clusters of differentiation 86 (CD86), Arginase 1 (Arg-1) and clusters of differentiation 206 (CD206). Cell viability and apoptosis were analyzed by MTT assay and TUNEL assay. mRNA levels of tumor necrosis factor-α (TNF-α), interleukin-1β (IL-1β), interleukin-4 (IL-4) and tumor necrosis factor-β (TNF-β) were quantified by quantitative real-time polymerase chain reaction. The association of SP1 and HTR2B was identified by chromatin immunoprecipitation assay and dual-luciferase reporter assay. HE staining assay was performed to analyze the pathological conditions of spinal cord tissues.

**Results:**

LPS treatment induced cell apoptosis and inhibited microglia polarization from M1 to M2 phenotype, accompanied by an increase of Bax protein expression and a decrease of Bcl-2 protein expression, however, these effects were relieved after SP1 silencing. Mechanism assays revealed that SP1 transcriptionally activated HTR2B in BV2 cells, and HTR2B knockdown rescued LPS-induced effects on BV2 cell apoptosis and microglial M1/M2 polarization. Moreover, SP1 absence inhibited BV2 cell apoptosis and promoted microglia polarization from M1 to M2 phenotype by decreasing HTR2B expression. SCI mouse model assay further showed that SP1 downregulation could attenuate spinal hitting-induced promoting effects on cell apoptosis of spinal cord tissues and microglial M1 polarization.

**Conclusion:**

SP1 transcriptionally activated HTR2B to aggravate traumatic SCI by shifting microglial M1/M2 polarization.

## Introduction

Spinal cord injury (SCI), as the name suggests, refers to damage to the spinal cord, which is a crucial part of the central nervous system responsible for controlling movement and sensation in the body. It can lead to impairment in function and sensation in the affected area, as well as in parts of the body below the injury site. This injury can have a significant impact on an individual’s life, resulting in loss of mobility, difficulties with bowel and bladder control, and persistent pain [1]. Macrophages are specialized immune cells that play a critical role in the immune response in various diseases such as musculoskeletal ailments, renal diseases and SCI [2–5]. They are divided into two major types: M1 and M2 macrophages, and the balance between M1 and M2 macrophages is crucial for optimal healing and functional recovery after SCI [6]. Research has shown that an imbalance in macrophage polarization can contribute to the persistence of inflammation, neuropathic pain, and poor functional outcomes [7, 8]. Therefore, modulating macrophage polarization becomes an important strategy in the treatment and management of SCI. However, the mechanism of macrophage polarization after SCI remains unclear.

Trans-acting transcription factor 1, also known as SP1, is a key protein involved in regulating gene expression. SP1 binds to specific DNA sequences and controls the transcription of various target genes [9]. In humans, SP1 plays a critical role in various biological processes, including cell growth, development, and differentiation [10]. Specifically, SP1 has been shown to regulate the expression of neurotrophic factors, such as brain-derived neurotrophic factor (BDNF), which are crucial for neuronal survival and regeneration [11]. SP1 has been extensively studied and found to have diverse roles in human diseases, particularly in spinal cord injury [12, 13]. Studies have shown that SP1 can modulate neuroinflammation and cellular apoptosis following SCI [14]. Understanding the precise role and mechanism of SP1 in SCI are crucial for developing targeted therapeutic strategies.

5-Hydroxytryptamine (serotonin) receptor 2B, also known as HTR2B, is a type of receptor for the neurotransmitter serotonin (5-HT) [15]. Serotonin is a key signaling molecule in the brain and plays a crucial role in regulating various physiological and behavioral processes, including mood, sleep, and appetite. In addition to its role in normal physiological functions, HTR2B has been associated with various human diseases. It has been extensively studied to understand its involvement in conditions such as cardiovascular diseases, psychiatric disorders, and cancer [15–17]. In particular, HTR2B has been found to promote M1 microglia polarization as well as inflammation following SCI through regulation of neuregulin-1/ErbB pathway [18].

We found that SP1 potentially bound to the promoter region of the HTR2B gene through the JASPAR online database, indicating that SP1 might transcriptionally activate HTR2B to modulate SCI. However, whether HTR2B is involved in the regulation of SP1 in spinal cord injury has not been reported. Further research is needed to reveal the interactions between these molecules in spinal cord injury. Understanding these mechanisms can provide new targets for the treatment of spinal cord injury, thereby improving patient outcomes.

## Materials and methods

### Microglia culture and treatment

Mouse microglia (BV2) was purchased from EK-Bioscience (Shanghai, China) and cultured in DMEM (Oumarsi Biotech, Shanghai, China) supplemented with 10% FBS and 1% penicillin/streptomycin (Cytiva, Shanghai, China) at 37℃ with 5% CO_2_. BV2 cells were exposed to lipopolysaccharide (1 µg/mL, Abmole Bioscience, Shanghai, China) for 24 h to investigate macrophage polarization, as instructed [19].

### Cell transfection

Ribobio Co., Ltd. (Guangzhou, China) synthesized small hairpin RNAs of SP1 (sh-SP1, Accession, NM_013672.2, 5’-CCAACTTACAGAACCAGCAAGTTCT-3’) and HTR2B (sh-THR2B, Accession: NM_008311.3, 5’-GATCCTGACTAACCGTTCTGGATTA-3’). Coding sequence of SP1 (101 to 2446 bp of the SP1 gene) was amplified by polymerase chain reaction (PCR) using specific primers with restriction sites and then inserted into the pcDNA 3.1 vector (Genomeditech, Shanghai, China) to establish SP1 overexpression plasmid (oe-SP1). Similarly, the HTR2B gene was amplified by PCR from 337 to 1776 bp, and the resulting fragment was used to construct an overexpression plasmid for HTR2B (oe-HTR2B) using pcDNA 3.1 vector. The day before transfection, the BV2 cells were passaged at a suitable density and allowed to reach a density between 50 and 70%. This ensures that the cells are in the logarithmic growth phase when they are transfected. Cells were transferred into 6-well plates with 1600 µL of DMEM without serum. The oligonucleotides, vectors, and transfection reagent LipoFiter (Hanbio, Shanghai, China) were mixed with the culture medium separately and allowed to incubate at room temperature for 5 min. The plasmid and liposomes were gently mixed together and then directly added to the cells in 6-well plates. The plates were then transferred to the incubator for culturing for 48 h.

### Western blotting assay

The processed cells were gently scraped and collected in centrifuge tubes. The spinal cord tissues of each mouse were cut with scissors and placed in centrifuge tubes. Each tube was then added with 300 µL of cell lysis buffer RIPA (Beyotime, Shanghai, China) and thoroughly lysed on ice. After lysis, the protein concentration of each sample was adjusted to the same level using sterile ddH_2_O based on the measured protein concentration. An equivalent volume of 5× SDS-PAGE protein loading buffer (Thermo Fisher, Waltham, MA, USA) was added to each sample and incubated in a boiling water bath for 10 min. The electrophoresis tank was filled with electrophoresis buffer, and the samples were quantitated and loaded onto the gel. After bromophenol blue reached the gel bottom, electroblotting was performed. The PVDF membrane obtained after transfer was immersed in skim milk solution prepared in TBST. Subsequently, the membrane was incubated with the primary antibodies against SP1 (#AF6121, 1:1000, Affinity, Nanjing, China), HTR2B (#DF3500, 1:1000, Affinity), BCL2-associated x protein (Bax, #AF0120, 1:1000, Affinity), B-cell lymphoma-2 (Bcl-2, #AF6139, 1:1000, Affinity), inducible nitric oxide synthase (iNOS, #AF0199, 1:1000, Affinity), clusters of differentiation 86 (CD86, #DF6332), Arginase 1 (Arg-1, #DF6657, 1:1000, Affinity) and clusters of differentiation 206 (CD206, #DF4149, 1:1000, Affinity) overnight on a rocking shaker at 4 °C, followed by incubation with a secondary antibody (#S0001, 1:5000, Affinity) on the second day. The chemiluminescent substrate was evenly applied to the protein side of the membrane and visualized using a gel imaging system before capturing the image.

### 3-(4,5-dimethylthazol-2-yl)-2,5-diphenyltetrazolium bromide (MTT) assay

25 mg MTT (Beyotime) was dissolved in 5 mL of dissolving solution, and cell culture was performed in 96-well plates. When the cell confluence reached 70–80%, cell transfection and LPS stimulation were conducted. After 48 h of culture, MTT with a final concentration of 2 µg/mL was added into each well. After 4 h of culture, the cells in each well were analyzed using an enzyme-linked immune detector.

### TUNEL cell apoptosis assay

Cell suspensions were prepared at a concentration of approximately 2 × 10^7^ cells/mL in PBS and pipetted onto glass slides coated with poly-L-lysine. The slides were immersed in a staining jar containing 4% fresh polyformaldehyde (Solarbio, Beijing, China) dissolved in PBS, and 100 µL of Proteinase K (Solarbio) was applied to each sample. To analyze cell apoptosis of spinal cord tissues, the tissues were embedded into paraffin and incubated with xylene, ethanol and Proteinase K. Subsequently, 100 µL of 1×Equilibration Buffer was added to cover the entire area of the samples, and TdT incubation buffer was added to the cells. The slides were then placed in a dark staining jar containing a solution of propidium iodide (PI, Solarbio). Excess water was removed from the slides by tapping, and 100 µL of PBS was added to maintain sample moisture. Finally, the samples were analyzed immediately under a fluorescence microscope.

### Quantitative real-time PCR (qRT-PCR)

Frozen cells and tissues were allowed to fully dissolve at room temperature for 5 min. 1/5 volume of chloroform to TRIZOL reagent was added, and the aqueous phase was transferred to clean centrifuge tubes free of RNase and mixed with an equal volume of isopropanol to precipitate the RNA. The samples were incubated with 75% ethanol, and the RNA was allowed to dry at room temperature. Subsequently, the obtained RNA was mixed with the primers from the cDNA synthesis kit (Wanleibio, Shenyang, China) and incubated at 70 °C for 5 min, followed by incubation for 5 min on ice. Then, the mixtures were incubated with the M-MLV reverse transcriptase and dNTPs from the cDNA synthesis kit (Wanleibio) at 42 °C for 50 min, and incubated at 80 °C for 10 min to synthesize cDNA. Finally, gene expression was quantified using SYBR Green reagent (TaKaRa, Dalian, China) according to the guidebook and analyzed through the 2^−∆∆Ct^ method. Primer sequences are shown in Table 1.

**Table 1:** Primers sequences used for PCR

Name		Primers for PCR (5’-3’)
SP1	Forward	GTCAGCGTCCGCGTTTTTC
	Reverse	CGCTACCCCCATTATTGCCA
HTR2B	Forward	CTCAGAGCAAGTCAGTGGGG
	Reverse	TGTGTACACGTCTGTCCGTG
β-actin	Forward	GAGCGCAAGTACTCTGTGTG
	Reverse	AACGCAGCTCAGTAACAGTCC

### Chromatin immunoprecipitation (ChIP) assay

According to the instruction manual of the assay kit (#WLA106a; Wanleibio), formaldehyde (Solarbio) was added to each sample and incubated at room temperature for 10 min. Glycine was incubated with the sample, and the medium was removed and washed twice with pre-chilled PBS. The cells were scraped into a 1.5 mL centrifuge tube using a cell scraper, centrifuged to collect the precipitate, and then incubated with Buffer A and Buffer B, as well as EDTA. After terminating the digestion for two min on ice, the precipitate was collected by centrifugation. The nuclear membrane was disrupted using an ultrasound bath, and anti-SP1 antibody (#MA5-35331, 1:100, Thermo Fisher) was added to the samples, which were then incubated overnight at 4 °C on a rocking mixer. The sample was then mixed with magnetic beads, allowing the supernatant to be removed by a magnetic separator. ChIP Elution Buffer was added to the magnetic beads, which were placed at 4 °C for half an hour. After DNA purification, HTR2B expression was analyzed using qRT-PCR.

### Dual-luciferase reporter assay

Upon accessing the “JASPAR” website (http://jaspar.genereg.net/), users entered “SP1” into the search bar and chose the SP1 ID number (MA0079.2) specific to the mouse species when it appeared in the search results. Subsequently, the promoter sequence of HTR2B was inputted into the scanning field, and upon clicking the “Scan” button, the binding sites would be displayed. To view the SP1 sequence logo, users could simply click on the SP1 ID number. The luciferase plasmids of HTR2B were prepared using pGL3-Basic vector and named as site-wt and site-mut. The reporter plasmid containing both the firefly luciferase and Renilla luciferase genes was transfected into each well (24-well plate) at a ratio of 50:1. A Dual-Luciferase Assay kit (Solarbio) was used to analyze luciferase activity according to the instructions. Each well was added with 1x PLB lysis buffer and agitated at room temperature for 15 min. Finally, the samples were detected using an automated luminescence detection instrument.

### SCI mouse model

Male adult mice, weighting 20–25 g, were purchased from Hunan Slyke Jingda Experimental Animal Co., LTD (Changsha, China) and housed in experimental animal center. The mice were anesthetized by intraperitoneal injection of chloral hydrate to ensure they were unable to feel pain during the injury procedure. Skin disinfection was conducted and T9-T11 spinous processes were exposed by making a longitudinal incision on the back. Then, T9-T10 laminas were removed and SCI was established by hitting. The retraction of the hind legs and the wagging of the tail in mice indicated that the SCI mouse model has established successfully. The above incisions were sutured layer by layer. Mice in the sham group, only the laminae and spinous processes were removed. Subdural injection of corresponding lentivirus expressing sh-SP1 (FulenGen, Guangzhou, China) or sh-NC (FulenGen) was performed at 5 min after SCI. According to the above methods, the mice were divided into the sham group, the SCI + sh-NC group (referred to as the SCI group in the study), and the SCI + sh-SP1 group. The study was approved by the Animal Care and Use Committee of the First People’s Hospital of Pingdingshan. Animal studies were performed in compliance with the ARRIVE guidelines and the Basel Declaration.

### Locomotor function recovery assessment

The Basso Mouse Scale (BMS) was used to analyze neurological function at the defined time points (1, 3, 7, 14, 21 and 28 day following SCI) according to the published method [20]. In addition, all mice were evaluated with an accelerated rotating rod (0–40 rpm) twice, 20 min apart, to assess capability and coordination of mice by evaluating the speed and duration of each mouse after spinal cord injury.

### Haematoxylin and eosin (HE) staining

The spinal cord was removed from all mice, and the excess tissues around the spinal cord were trimmed and rinsed using PBS to remove any debris or blood. The spinal cord was fixed in 10% neutral buffered formalin (Solarbio) and dehydrated using alcohol (Solarbio). The dehydrated tissues were transferred to xylene for complete clearance and embedded in liquid paraffin. The paraffin-embedded blocks were cut using a microtome and transferred to glass slides carefully. Deparaffinization and rehydration were then performed, and the rehydrated sections were immersed in hematoxylin solution (Solarbio) and counterstained using eosin solution (Solarbio). The sections were gradually dehydrated by passing them through increasing alcohol concentrations and observed under a light microscope.

### Statistical analysis

GraphPad Prism was used to analyze all data from three independent biological replicates, and results were shown as means ± standard deviations (SD). Significant differences were compared using Student’s *t*-tests, one-way analysis of variance or two-way analysis of variance. *P* < 0.05 indicated statistical significance.

## Results

### SP1 silencing promoted microglia polarization from M1 to M2 phenotype after LPS treatment in vitro

**Fig. 1:**
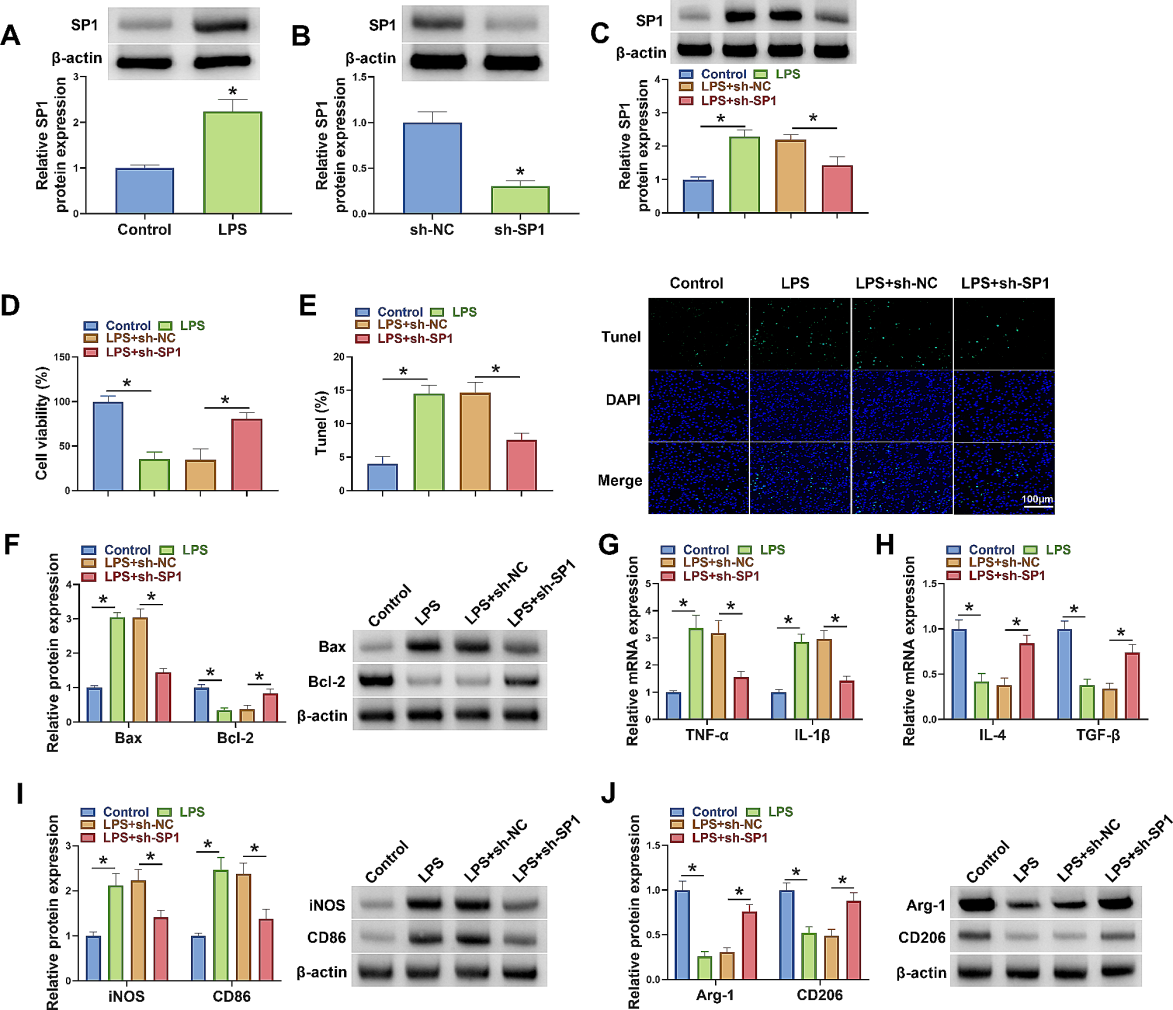
SP1 knockdown promoted the polarization of LPS-induced BV2 microglia from M1 to M2 phenotype in vitro. (**A**) The effect of LPS treatment on SP1 protein expression was analyzed by western blotting assay. (**B**) The effect of SP1 shRNA in downregulating SP1 protein expression was determined by western blotting assay. (**C**-**J**) BV2 cells were divided into control group, LPS group, LPS + sh-NC group, and LPS + sh-SP1 group. (**C**) SP1 protein expression was analyzed by western blotting assay. (**D**) MTT assay was used to analyze cell viability. (**E**) Tunel assay was performed to analyze cell apoptosis. (**F**) Western blotting assay was used to detect protein expression of Bax and Bcl-2. (**G** and **H**) qRT-PCR assays were conducted to analyze the mRNA expression levels of TNF-α, IL-1β, IL-4 and TGF-β. (**I** and **J**) iNOS, CD86, Arg-1 and CD206 protein levels were analyzed by western blotting assay. *P < 0.05

The study first analyzed the effects of SP1 silencing on biological behaviors of LPS-induced BV2 cells, including cell viability, cell apoptosis, and BV2 microglia polarization. As shown in Fig. 1A, LPS treatment increased SP1 protein expression. The high efficiency of SP1 shRNA in downregulating SP1 expression was analyzed by western blotting assay, and the results are shown in Fig. 1B. Subsequently, the results showed that LPS-induced an increase of SP1 protein expression was attenuated after SP1 silencing (Fig. 1C). LPS treatment inhibited cell viability and induced cell apoptosis, accompanied by an increase of Bax protein expression and a decrease of Bcl-2 protein expression, however, these effects were rescued after transfection with shRNA of SP1 (Fig. 1D-F). Additionally, the results revealed that LPS treatment promoted TNF-α and IL-1β mRNA levels but inhibited IL-4 and TGF-β mRNA levels, whereas these effects were relieved after SP1 knockdown (Fig. 1G and H). Similarly, LPS-induced cells showed increased iNOS and CD86 protein expression and decreased Arg-1 and CD206 protein expression, but these effects were rescued after SP1 silencing (Fig. 1I and J). Therefore, SP1 knockdown promoted the polarization of LPS-induced BV2 microglia from M1 to M2 phenotype.

### SP1 transcriptionally activated HTR2B in BV2 cells

**Fig. 2:**
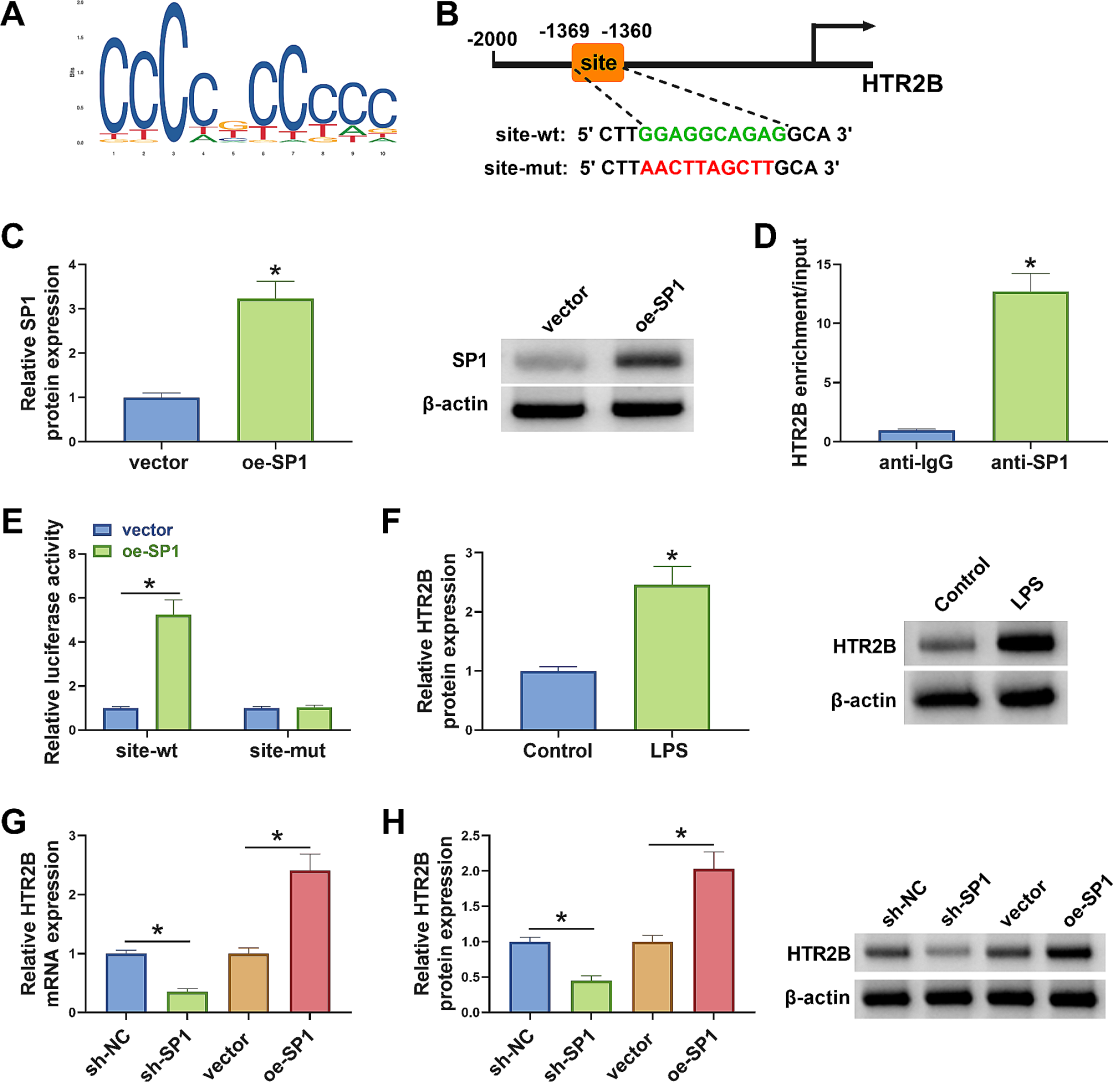
SP1 transcriptionally activated HTR2B in BV2 cells. (**A**) DNA binding motif of SP1 was obtained from the JASPAR online database. (**B**) The schematic illustration showed the binding sites of SP1 for HTR2B. (**C**) Western blotting assay was used to detect SP1 protein expression after transfection with vector or SP1 overexpression plasmid. (**D** and **E**) ChIP assay and dual-luciferase reporter assay were conducted to identify the association of SP1 and HTR2B. (**F**) The effect of LPS treatment on HTR2B protein expression was analyzed by western blotting assay. (**G** and **H**) Western blotting assay and qRT-PCR assay were performed to analyze the effects of abnormal SP1 expression on HTR2B expression. *P < 0.05

The study continued to analyze the downstream target of SP1 in BV2 cells. We found that SP1 potentially bound to the promoter region of the HTR2B gene, as predicted through the JASPAR online database (Fig. 2A and B). Subsequently, the results identified the association of SP1 and HTR2B using ChIP and dual-luciferase reporter assays. The results from Fig. 2C showed high efficiency of SP1 overexpression plasmid in increasing SP1 protein expression in BV2 cells. As shown in Fig. 2D, SP1 had a strong affinity in the promoter region of HTR2B. Dual-luciferase reporter assay showed that SP1 overexpression promoted luciferase activity of wild-type HTR2B but had no significant effect on luciferase activity of mutant HTR2B (Fig. 2E). Additionally, the results showed that LPS treatment increased HTR2B protein expression (Fig. 2F). Further, mRNA and protein expression of HTR2B were inhibited after SP1 silencing but promoted after SP1 overexpression (Fig. 2G and H). Thus, HTR2B could be transcriptionally activated by SP1 in BV2 cells.

### HTR2B silencing promoted the polarization of LPS-induced BV2 microglia from M1 to M2

**Fig. 3:**
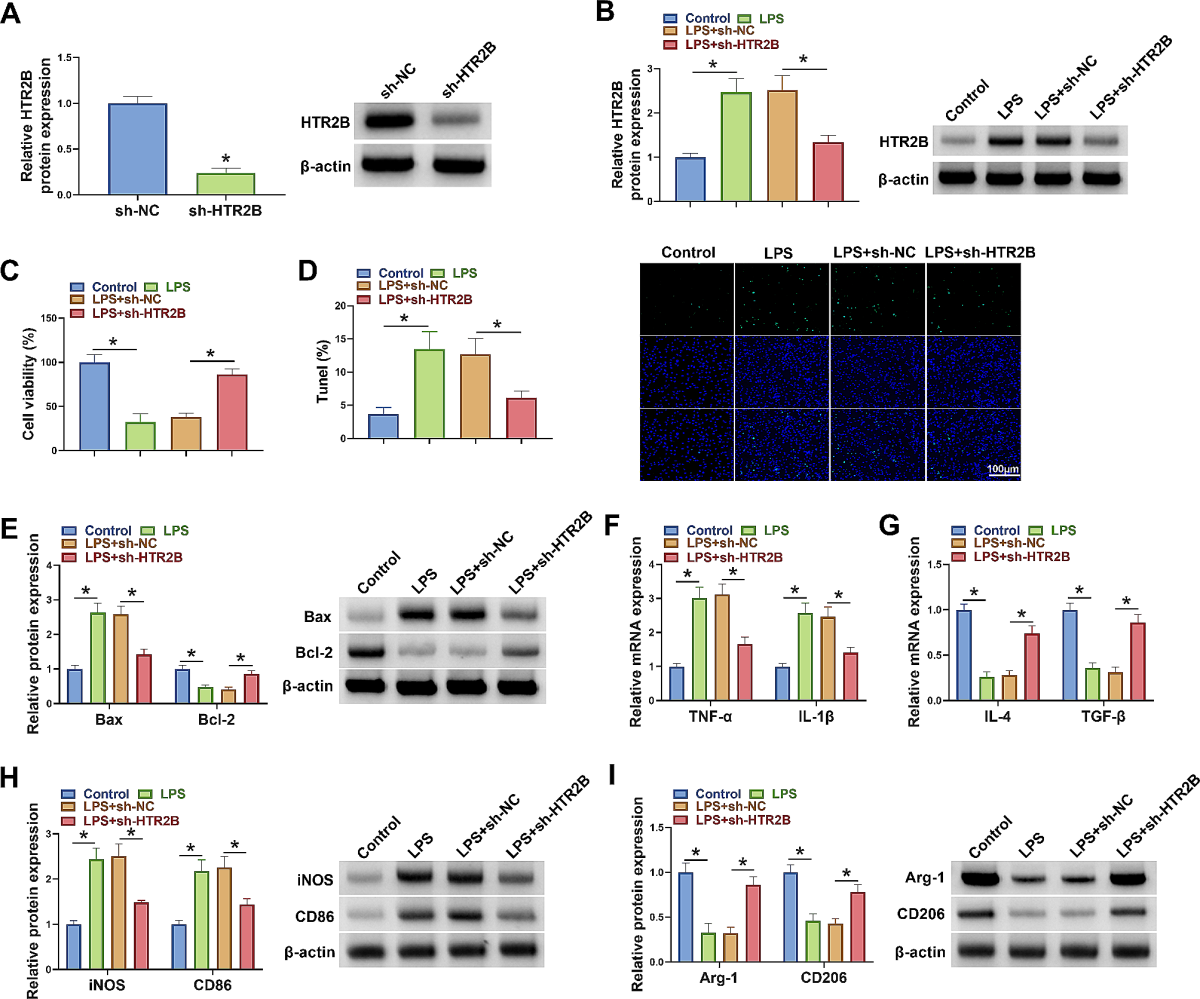
HTR2B knockdown promoted the polarization of LPS-induced BV2 microglia from M1 to M2. (**A**) The effect of HTR2B shRNA in downregulating HTR2B protein expression was determined by western blotting assay. (B-I) BV2 cells were divided into control group, LPS group, LPS + sh-NC group, and LPS + sh-HTR2B group. (**B**) HTR2B protein expression was determined by western blotting assay. (**C**) MTT assay was used to analyze cell viability. (**D**) Tunel assay was performed to analyze cell apoptosis. (**E**) Western blotting assay was used to detect protein expression of Bax and Bcl-2. (**F** and **G**) qRT-PCR assays were conducted to analyze the mRNA expression levels of TNF-α, IL-1β, IL-4 and TGF-β. (**H** and **I**) iNOS, CD86, Arg-1 and CD206 protein levels were analyzed by western blotting assay. *P < 0.05

The study further analyzed the effects of HTR2B silencing on biological behaviors of LPS-induced BV2 cells. The high efficiency of HTR2B shRNA in downregulating HTR2B expression was analyzed by western blotting assay, and the results are shown in Fig. 3A. Subsequently, the results showed that LPS-induced an increase of HTR2B protein expression was attenuated after HTR2B silencing (Fig. 3B). LPS treatment inhibited cell viability and induced cell apoptosis, accompanied by an increase of Bax protein expression and a decrease of Bcl-2 protein expression, however, these effects were rescued after transfection with shRNA of HTR2B (Fig. 3C-E). Additionally, the results revealed that LPS treatment promoted TNF-α and IL-1β mRNA levels and inhibited IL-4 and TGF-β mRNA levels, whereas these effects were relieved after HTR2B knockdown (Fig. 3F and G). Similarly, LPS-induced cells showed increased iNOS and CD86 protein expression and decreased Arg-1 and CD206 protein expression, but these effects were rescued after HTR2B silencing (Fig. 3H and I). Therefore, HTR2B knockdown promoted the polarization of LPS-induced BV2 microglia from M1 to M2.

### HTR2B overexpression attenuated SP1 silencing-induced effects on microglia M1/M2 polarization in LPS-stimulated BV2 cells

**Fig. 4:**
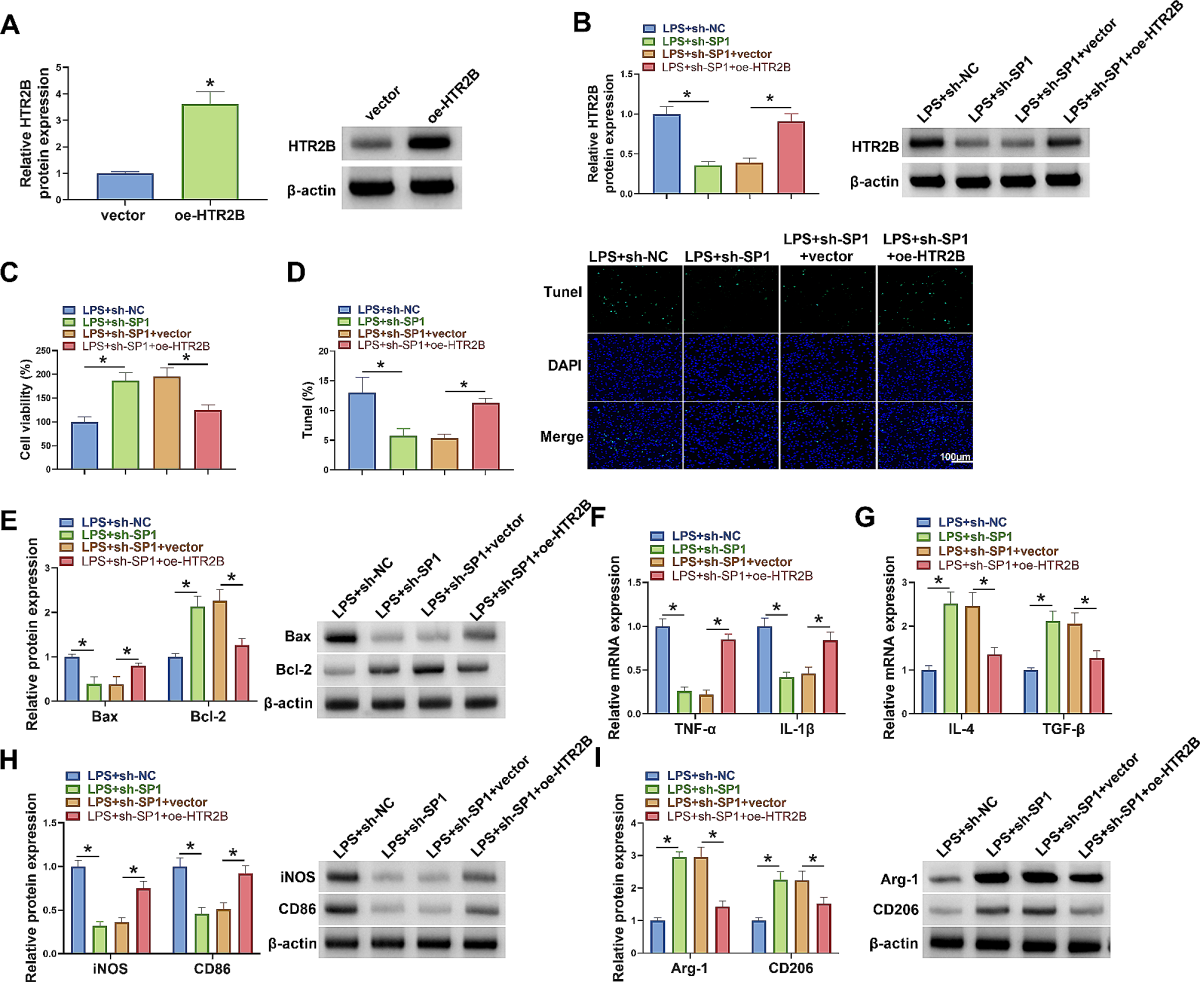
SP1 silencing promoted polarization of microglia M2 phenotype by decreasing HTR2B expression. (**A**) Western blotting assay was used to detect HTR2B protein expression in BV2 cells transfected with vector or oe-HTR2B. (B-I) BV2 cells were divided into LPS + sh-NC group, LPS + sh-SP1 group, LPS + sh-SP1 + vector group and LPS + sh-SP1 + oe-HTR2B group. (**B**) HTR2B protein expression was determined by western blotting assay. (**C**) MTT assay was used to analyze cell viability. (**D**) Tunel assay was performed to analyze cell apoptosis. (**E**) Western blotting assay was used to detect protein expression of Bax and Bcl-2. (**F** and **G**) qRT-PCR assays were conducted to analyze the mRNA expression levels of TNF-α, IL-1β, IL-4 and TGF-β. (**H** and **I**) iNOS, CD86, Arg-1 and CD206 protein levels were analyzed by western blotting assay. *P < 0.05

Whether HTR2B was involved in the regulation of SP1 in biological behaviors of LPS-induced BV2 cells was analyzed in this part. To this end, the study co-transected SP1 shRNA and HTR2B overexpression plasmid into BV2 cells, followed by treatment with LPS. The high efficiency of HTR2B overexpression plasmid in increasing HTR2B protein expression was confirmed by western blotting assay, as revealed by increased HTR2B protein expression after transfection (Fig. 4A). Subsequently, SP1 silencing inhibited HTR2B protein expression in LPS-induced BV2 cells, but the effect was attenuated after HTR2B overexpression (Fig. 4B). As shown in Fig. 4C-E, SP1 knockdown promoted cell viability and inhibited cell apoptosis, accompanied by a decrease of Bax protein expression and an increase of Bcl-2 protein expression, however, these effects were rescued after HTR2B overexpression. The results also revealed that SP1 absence led to decreased TNF-α, IL-1β, iNOS and CD86 expression and increased IL-4, TGF-β, Arg-1 and CD206 expression in LPS-induced BV2 cells, whereas these effects were rescued after HTR2B overexpression (Fig. 4F-I). The above data demonstrated that SP1 silencing promoted microglia M2 polarization by decreasing HTR2B expression.

### SP1 silencing ameliorated SCI and promoted the polarization of LPS-induced microglia from M1 to M2 phenotype in vivo

**Fig. 5:**
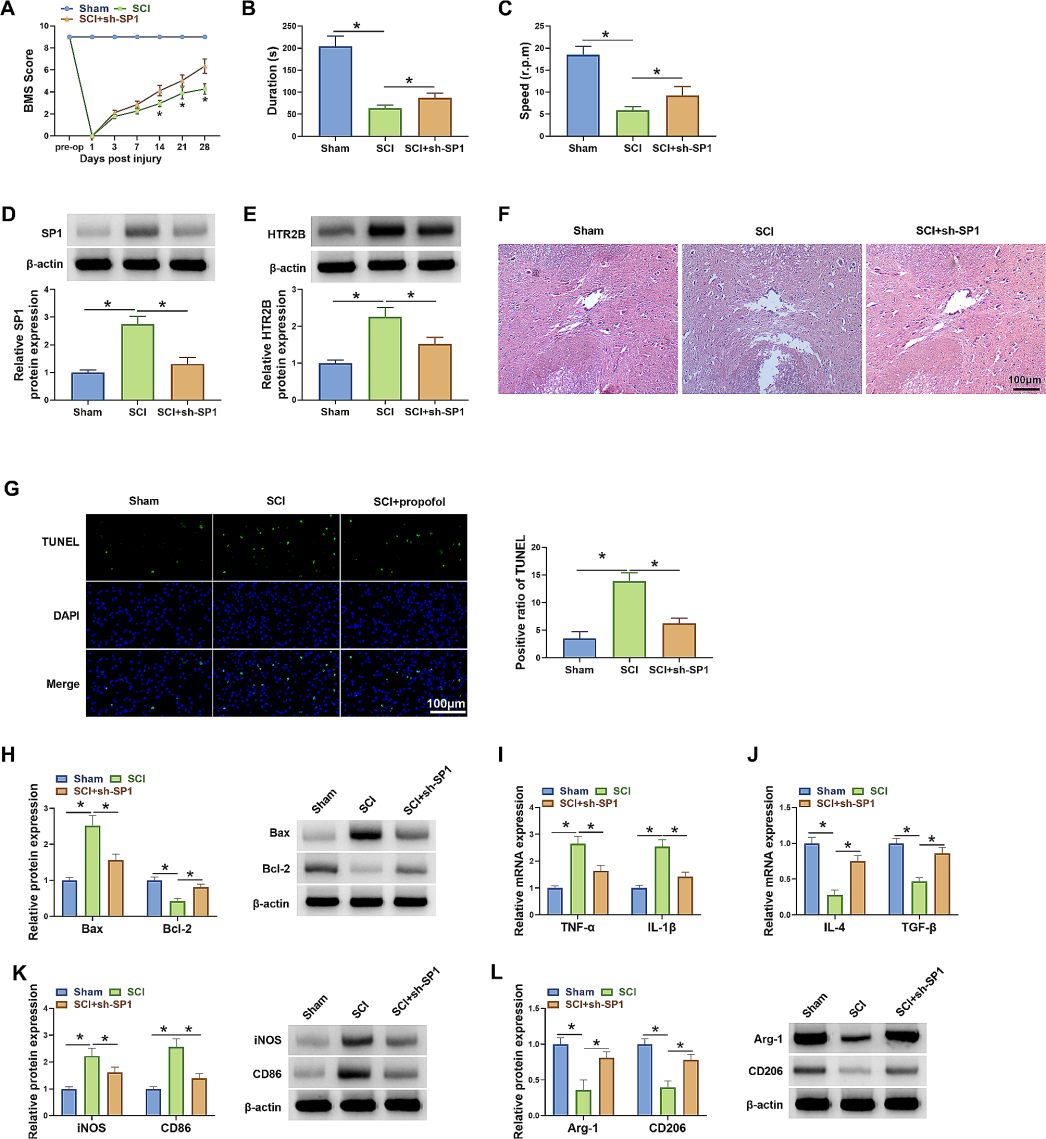
SP1 knockdown ameliorated SCI and contributed to the polarization of LPS-induced BV2 microglia from M1 to M2 phenotype in vivo. SCI mouse model was established by hitting from a height of 3 cm and subdural injection of the corresponding lentivirus expressing sh-SP1 in the lesioned area was conducted 5 min after spinal hitting. (**A**) Basso Mouse Scale (BMS) score was used to functionally grade the mice in the Sham group, SCI group and SCI + sh-SP1 group. (**B** and **C**) Rotarod test was used to analyze motor function of each mice. (**D** and **E**) Western blotting assay was used to detect SP1 and HTR2B protein expression in the spinal cord tissues of each mouse. (**F**) HE staining assay was performed to analyze the pathological conditions of spinal cord tissues of each mouse. (**G**) Cell apoptosis of spinal cord tissues of each mouse was analyzed by TUNEL assay. (**H**) Western blotting assay was used to detect Bax and Bcl-2 protein expression. (**I** and **J**) qRT-PCR was performed to detect mRNA expression levels of TNF-α, IL-1β, IL-4 and TGF-β in the spinal cord tissues of each mouse. (**K** and **L**) iNOS, CD86, Arg-1 and CD206 protein levels were analyzed by western blotting assay in the spinal cord tissues of each mouse. *P < 0.05

To reveal the regulatory role of SP1 silencing in microglia polarization in vivo, we established a SCI mouse model. The functional recovery of mice treated with SCI and/or lentivirus expressing sh-SP1 was analyzed through the BMS. As shown in Fig. 5A, mice in the SCI + sh-SP1 group showed better functional improvement when compared with the mice in the SCI group, as indicated by a higher BMS score in the defined time points. The results of the rotarod test (28 days post-SCI) also implied better recovery of motor function in the SCI + sh-SP1 group than in the SCI group (Fig. 5B and C). Additionally, the results showed that SP1 and HTR2B protein expressions were increased in the spinal cord tissues after SCI, whereas the effect was attenuated after SP1 silencing (Fig. 5D and E). HE staining assay showed that alleviated tissue injury and less neuronal loss (characterised by damaged nuclei and shrunken cytoplasm) in the SCI + sh-SP1 group than in the SCI group (Fig. 5F). Moreover, the results showed that SCI treatment induced cell apoptosis, increased Bax protein expression and decreased Bcl-2 protein expression in the spinal cord tissues after SCI, however, these effects were attenuated after SP1 absence (Fig. 5G and H). Further, SCI treatment increased TNF-α, IL-1β, iNOS and CD86 expression and decreased IL-4, TGF-β, Arg-1 and CD20 expression in the spinal cord tissues, whereas these effects were relieved after SP1 deficiency (Fig. 5I-L). Thus, SP1 knockdown contributed to the polarization of LPS-induced BV2 microglia from M1 to M2 phenotype in vivo.

## Discussion

Spinal cord injury can occur due to various causes such as trauma, infection, or degenerative diseases. Following the injury, there is an immediate immune response characterized by activation of immune cells, release of inflammatory mediators, and recruitment of immune cells to the site of damage [21]. Macrophages are specialized immune cells and can be M1 (classically activated) or M2 (alternatively activated) phenotype, depending on the signals they receive. M1 macrophages are involved in pro-inflammatory responses and pathogen elimination, while M2 macrophages play a role in tissue repair, wound healing, and anti-inflammatory processes [22]. The polarization of macrophages in spinal cord injury is a dynamic process that influences the pathogenesis and outcome of the injury. However, the specific mechanism remains unclear. The present work revealed that SP1 transcriptionally activated HTR2B to aggravate traumatic SCI by shifting microglial M1/M2 polarization.

Previous work has revealed that SP1 knockdown inhibited astrocyte proliferation and migration in a SCI cell model [23]. Additionally, SP1 could promote apoptosis of H_2_O_2_-induced PC12 cells, a SCI cell model [24]. SP1 was highly expressed in spinal cord tissues of SCI rat and its downregulation ameliorated cell apoptosis of ischemia/reperfusion-induced spinal cord tissues [14].We analyzed the role of SP1 in SCI using both LPS-induced BV2 cells and mice with SCI. The results showed that SP1 protein expression was upregulated in both LPS-induced BV2 cells and SCI mouse model. SP1 silencing reversed cell apoptosis induced by LPS and spinal hitting. M1 macrophages typically express high levels of pro-inflammatory cytokines such as TNF-α and IL-1β, while M2 macrophages typically express high levels of anti-inflammatory cytokines such as IL-4 and TGF-β [25]. iNOS is an enzyme that catalyzes the production of nitric oxide, which plays a crucial role in immune response and is expressed on M1 macrophages [26]. CD86 is a surface receptor that promotes immune cell activation and is expressed on M1 macrophages [26]. Arg-1 is an enzyme that catalyzes the conversion of arginine to polyamines, which are essential for cell growth and proliferation and is expressed on M2 macrophages [27]. CD206 is a surface receptor that promotes immune cell activation and is expressed on M2 macrophages [27]. Our evaluated the effects of SP1 on macrophage polarization after SCI by analyzing these cytokines and proteins. The results showed that SP1 silencing inhibited TNF-α, IL-1β, iNOS and CD88 expression and promoted IL-4, TGF-β, Arg-1 and CD206 in both LPS-induced BV2 cells and mice with SCI, indicating that SP1 promoted microglia M1 polarization.

As a transcription factor, SP1 binds to specific DNA sequences and promotes the transcription of target genes in human diseases. For example, SP1 transcriptionally activated long non-coding RNA THAP7-AS1 to mediate gastric cancer progression [28]. SP1 transcriptionally activated NLRP6 to induce radioresistance in glioma cells [29]. Our results revealed that HTR2B was transcriptionally activated by SP1 in BV2 cells and HTR2B silencing attenuated LPS-induced BV2 cell apoptosis and microglia M1 polarization. Moreover, HTR2B overexpression relieved SP1 silencing-induced effects in LPS-stimulated BV2 cells. A previous study has revealed that HTR2B expression was upregulated in lipopolysaccharide-stimulated microglia and SCI rats [18]. Similarly, our data showed that LPS-induced BV2 cells and SCI mice showed a high HTR2B expression. It has been reported that HTR2B inhibits the inactivation of Nrg-1/ErbB signaling to promote M1 microglia polarization and neuroinflammation following spinal cord injury [18]. HTR2B can activate specific signaling pathways such as the PI3K/Akt pathway, which has been shown to enhance cell survival and inhibit apoptosis [15]. Additionally, hydrogen peroxide exposure can induce oxidative damage, accompanied by a decrease in HTR2B expression [30], which suggests that HTR2B may inhibit oxidative stress, a trigger for apoptosis. Thus, HTR2B may activate PI3K/Akt pathway and reduce oxidative stress to protect microglia from apoptosis. These results demonstrated that HTR2B was involved in the regulation of SP1 in M1 microglia polarization and microglia apoptosis after SCI.

Thus, SP1 transcriptionally activated HTR2B to promote microglia M1 polarization and microglia apoptosis after SCI. Modulating the macrophage phenotype towards an anti-inflammatory and tissue repair-promoting state may hold promise for developing therapeutic strategies to enhance recovery and functional outcomes in spinal cord injury. However, there are several limitations in using mouse-derived cells and mouse models to study the role and mechanism of SP1 in SCI. Mouse-derived cells and human cells have differences in genomics, proteins, and phenotypes, which may lead to the irreproducibility of research results. In addition, the mouse model may not fully simulate the pathophysiological process of human SCI due to significant differences in the nervous systems between mice and humans. Thus, more accurate and human-relevant methods may help to better understand the microglia polarization and cell apoptosis processes after SCI.

## Data Availability

The data that support the findings of this study are available from the corresponding author upon reasonable request.
